# Seroprevalence and associated risk factors of *Toxoplasma gondii* infection in patients undergoing hemodialysis and healthy group

**DOI:** 10.1186/s13104-020-05396-5

**Published:** 2020-12-07

**Authors:** Shahrzad Soltani, Mehdi Sagha Kahvaz, Sheyda Soltani, Fatemeh Maghsoudi, Masoud Foroutan

**Affiliations:** USERN Office, Abadan Faculty of Medical Sciences, Abadan, Iran

**Keywords:** *Toxoplasma gondii*, Hemodialysis, Seroprevalence, ELISA, Iran

## Abstract

**Objectives:**

In this study, the seroprevalence of anti-*Toxoplasma gondii* (*T. gondii*) specific antibodies in patients undergoing hemodialysis compared to the control group were evaluated. In this case–control study, 200 hemodialysis patients (HDP) and 100 healthy controls were participated. The specific antibodies (IgG/IgM) in both groups were tested using enzyme-linked immunosorbent assay (ELISA) method. A structured questionnaire containing some demographic information was completed for each person in case and control groups.

**Results:**

The overall seroprevalence of *T. gondii* infection was 49.5% (99/200) and 23.0% (23/100) in the case and control groups, respectively. There was a significant association between seroprevalence of *T. gondii* infection and contact with cats (*P* < 0.001), consumption of raw/undercooked meat (*P* = 0.01), and source of drinking water (*P* = 0.001) in the hemodialysis patients. Also, in the control subjects, there were a significant association between consumption of raw/undercooked meat (*P* = 0.04) and source of drinking water (*P* = 0.001) with *T. gondii* infection. The findings showed a high seroprevalence of *T. gondii* infection in HDP compared with healthy controls; thus, we recommend the regular screening programs for *T. gondii* infection in this susceptible group.

## Introduction

*Toxoplasma gondii* (*T. gondii*) is the well-known intracellular parasite with widespread distribution in all continents which could infect a broad range of warm-blooded vertebrates [1, 2]. *T. gondii* infection is mainly transmitted through ingestion of oocyst-contaminated food or water, ingestion of raw/undercooked meat contaminated with tissue cysts, consumption of raw/unwashed vegetables, vertical transmission from mother to the fetus and rarely by organ transplantation and blood transfusion [3–7].

It is estimated that approximately one-third of the human population is chronically infected throughout the globe [3, 4, 8]. The previously articles with meta-analysis approach in Iran have reported the pooled seroprevalence rate of *T. gondii* infection in different human groups. For example, in some groups such as HIV-positive subjects, patients with malignancy, transplant recipients, general population, pregnant women, healthy blood donors, and hemodialysis patients (HDP), the seroprevalence rate was estimated to be 50%, 45%, 55%, 39%, 41%, 34%, and 58%, respectively [9–13].

Although, toxoplasmosis is sometimes asymptomatic in immunocompetent persons, it can cause severe complications or even may result in death in the immunocompromised subjects [3]. As it is evident, the patients undergoing hemodialysis are considered to be immunocompromised, mainly due to immune response dysfunctions regarding phagocytosis, chemotaxis, and the complement system [14]; thus, these subjects are more susceptible than healthy individuals to acquire several opportunistic microorganisms, such as *T. gondii* [13, 15]. The current investigation was aimed to evaluate the prevalence of anti-*T. gondii* specific antibodies in patients undergoing hemodialysis in comparison to control groups.

### Methods

In this case–control study, 200 HDP who were referred to Shahid Beheshti and Valiasr hospitals affiliated to the Abadan Faculty of Medical Sciences in the southwest Iran (Khuzestan Province, between 2018 and 2019) and 100 healthy controls were enrolled and venous blood samples were collected from all participants. The inclusion criteria were as follows: (1) subjects ≥ 10 years old; (2) volunteer to participate in the study upon obtaining a written informed consent; (3) in case group: the patients undergoing regular hemodialysis; and (4) in the control group: healthy volunteers with normal blood urea nitrogen and creatinine levels as well as without any renal disease. It should be noted that the subjects were matched in terms of gender and age in both groups.

A structured questionnaire containing some demographic information was developed and accomplished for each person in case and control groups, as previously described [16, 17]. The demographic information and related risk factors with *T. gondii* infection were as follows: gender, age, residence, education level, contact with cats, consumption of raw/undercooked meat, and source of drinking water.

From each participant who have met the inclusion criteria, 5 mL of venous blood was taken and then the samples were transferred to the laboratory of the Abadan Faculty of Medical Sciences. As earlier described [17], the sera were separated by centrifugation at 1700×*g* for 5 min and then stored in − 20 °C till examined. The specific antibodies (IgG/IgM) in both groups were measured using commercial available enzyme-linked immunosorbent assay kits (Torch-IgG, IgM-Trinity Biotech Company), according to the manufacturer’s guideline. The *Chi*-square test was performed using SPSS version 21 (SPSS Inc., Chicago, IL, USA). Also, we used Univariate logistic regression analysis to assess the probable association between the related risk factors and seropositivity of *T. gondii* infection. The level of significance was considered *P* < 0.05 [16].

## Results

The main characteristics and seroprevalence of *T. gondii* infection of the participants in case and control groups are listed in Table 1. Briefly, the overall seroprevalence of *T. gondii* infection was estimated to be 49.5% (99/200) and 23.0% (23/100) in the case and control groups, respectively. Of these, anti-*T. gondii* IgG antibodies were found in 40.0% (80/200) of HDP and 21.0% (21/100) of healthy controls, while IgM were identified in 9.5% (19/200) and 2.0% (2/100) of these groups, respectively (*P* < 0.05). Upon analysis, HDP were significantly more likely to be seropositive for IgG (OR, 2.50; 95% CI 1.43–4.38; *P* < 0.001) and IgM (OR, 5.14; 95% CI 1.17–22.54; *P* < 0.016) antibodies against *T. gondii* infection than healthy volunteers.

**Table 1:** Seroprevalence of *Toxoplasma gondii* infection in HDP and healthy controls [*n* (%)]

Characteristic	Hemodialysis patients					Control group				
	Number tested	Seropositive, n (%)	Univariate analysis			Number tested	Seropositive, n (%)	Univariate analysis		
			OR	95% CI	*P *value			OR	95% CI	*P *Value
Gender										
Male	105	54 (51.42)	0.85	0.48–1.48	0.56	53	12 (22.64)	1.04	0.41–2.65	0.92
Female	95	45 (47.36)	1.17	0.67–2.05		47	11 (23.40)	0.95	0.37–2.43	
Age										
≤ 20	15	7 (46.66)	1.00	Referent	0.99	10	1 (10.00)	1.00	Referent	0.99
21–40	35	16 (45.71)	1.039	0.30–3.49		19	4 (21.05)	0.75	0.07–8.08	
41–60	60	29 (48.33)	0.935	0.30–2.90		25	7 (28.00)	0.429	0.04–4.23	
61–80	76	40 (52.63)	0.788	0.26–2.38		37	8 (21.62)	0.604	0.06–5.77	
81–90	14	7 (50.00)	0.875	0.20–3.76		9	3 (33.33)	0.333	0.02–4.18	
Residence										
Urban	147	76 (51.70)	1.39	0.74–2.62	0.30	72	17 (23.61)	1.13	0.39–3.25	0.81
Rural	53	23 (43.39)	0.71	0.38–1.34		28	6 (21.42)	0.88	0.30–2.53	
Education level										
Diploma or lower	154	80 (51.94)	1.53	0.78–2.99	0.20	75	19 (25.33)	1.78	0.54–5.85	0.33
University degree	46	19 (41.30)	0.65	0.33–1.26		25	4 (16.00)	0.56	0.17–1.84	
Contact with cat										
Yes	70	49 (70.00)	3.73	2.00–6.95	< 0.001	33	10 (30.30)	1.80	0.69–4.70	0.22
No	130	50 (38.46)	0.26	0.14–0.49		67	13 (19.40)	0.55	0.21–1.44	
Consumption of raw/undercooked meat										
Yes	78	47 (60.25)	2.04	1.14–3.63	0.01	27	10 (37.03)	2.71	1.01–7.26	0.04
No	122	52 (42.62)	0.49	0.27–0.87		73	13 (17.80)	0.36	0.13–0.98	
Source of drinking water										
Sanitary water	145	61 (42.06)	0.32	0.16–0.62	0.001	83	14 (16.86)	0.18	0.05–0.54	0.001
Unsanitary water	55	38 (69.09)	3.07	1.59–5.95		17	9 (52.94)	5.54	1.82–16.86	

*HDP* hemodialysis patients, *OR* odds ratio, *CI* confidence interval

In this study, we have recorded seven probable risk factors associated with *T. gondii* infection. There was a significant association between seroprevalence of *T. gondii* infection and contact with cats (*OR* 3.73 [95% CI 2.00–6.95]; *P* < 0.001), consumption of raw/undercooked meat (*OR* 2.04 [95% CI 1.14–3.63]; *P* = 0.01), and source of drinking water (*OR* 3.07 [95% CI 1.59–5.95]; *P* = 0.001) in the hemodialysis patients. Also, in the control subjects, there were a significant association between consumption of raw/undercooked meat (*OR* 2.71 [95% CI 1.01–7.26]; *P* = 0.04) and source of drinking water (*OR* 5.54 [95% CI 1.82–16.86]; *P* = 0.001) with *T. gondii* infection. More details are shown in Table 1.

## Discussion

During the two past decades, an increasing trend was reported in the number of persons with renal failure and end-stage renal disease requiring hemodialysis [18]. There is a lack of knowledge about the epidemiological status of *T. gondii* infection in rural and urban communities of southwest Iran amongst patients undergoing hemodialysis. Thus, in the current study, we evaluated the seroprevalence of anti-*T. gondii* antibodies in HDP compared to control group from these regions. The results showed that the seroprevalence rate of *T. gondii* infection was higher in patients undergoing hemodialysis than normal subjects (49.5% vs. 23.0%). Our findings were in accordance with previous studies such as Ebrahim Zadeh in Zahedan city (Sistan and Baluchistan province) [19], Solhjoo et al. in Jahrom city (Fars province) [20], Bayani et al. in Babol city (Mazandaran province) [21], Saadat et al. in Rasht city (Guilan province) [22], Arefkhah et al. in Boyer-Ahmad city (Kohgiluyeh and Boyer-Ahmad province) [16], Saki et al. in Ahvaz city (Khuzestan province) [23], and Hamidi et al. in East Azerbaijan province [24].

In case group, 40.0% and 9.5% of HDP were found positive for IgG and IgM antibodies using ELISA, respectively. This seroprevalence rate of latent infection is similar with Khalili et al. study in Chaharmahal and Bakhtiari province (45.0%) [25]. In the majority of studies in different parts of Iran such as Tehran (60.0%) [26], Sistan and Baluchistan (73.7%) [27], East Azerbaijan (70.2%) [24], Guilan (72.0%) [22], Mazandaran (80.0%) [21], Isfahan and Qom provinces (63.0%) [28], the seropositivity in patients undergoing hemodialysis were reported over 60%, while the lower seroprevalence rate of *T. gondii* infection was reported by Saki et al. from Khuzestan province (29.3%) [23] and Arefkhah et al. from Kohgiluyeh and Boyer-Ahmad province (27.7%) [16]. The disagreement between studies is may be due to several reasons, including, study area, the number of participants, type of sampling, methodology, cultural habits of the subjects, different cutoff values or antibody titers and so on.

In a comprehensive systematic review with meta-analysis approach that performed by Foroutan et al. [13] in Iran, the pooled seroprevalence of latent and acute *T. gondii* infection in patients undergoing hemodialysis was evaluated up to December 2017. Finally, 1865 individuals (1048 HDP and 817 normal subjects as controls) were eligible to be included. The results revealed that 58% (95% CI 46–70%) and 40% (95% CI 31–50%) of HDP and healthy controls were seropositive in terms of IgG, while IgM antibody were detected in 2% (95% CI 0–6) and 0% (95% CI 0–1) of these groups, respectively. They concluded that patients undergoing hemodialysis were more likely to be seropositive for IgG (OR = 2.04; 95% CI 1.54–2.70; *P* < 0.001) and IgM (OR = 2.53; 95% CI 1.23–5.22; *P* < 0.001) antibodies against *Toxoplasma* infection than healthy volunteers. Also, the latent infection ranged from 29 to 80% with the highest prevalence in Mazandaran province [13]. Also, it is worth to mention that in two different systematic reviews among the Iranian general population (approximately 59%) and pregnant women (ranged from 56 to 75%), Mazandaran province revealed one of the highest seroprevalence rate of *T. gondii* infection throughout the country [10, 11]. It seems the climate status of this province is an important parameter. Mazandaran province has ideal mean humidity and annual rainfall, which considered as suitable conditions for *T. gondii* oocysts sporulation. Also, the cultural habits of the people and working on farming lands may be the other reasons [10].

The results of our study showed that there was a significant association between *T. gondii* seroprevalence and consumption of raw/undercooked meat and source of drinking water in both case and control groups. Belluco et al. [29] have confirmed that different meat products are considered as the main sources for acquiring the infection. So, they concluded that the increase in consumer knowledge definitely could influence in the reduction of the infection rate in societies [6, 29].

## Conclusions

In conclusion, our investigation showed a high seroprevalence of *T. gondii* infection in patients undergoing hemodialysis compared with healthy controls in southwest Iran. Since, these patients are immunocompromised and toxoplasmosis may cause severe and progressive complications with very poor prognosis in such patients, we recommend the regular screening programs for *T. gondii* infection into the routine clinical care of patients undergoing hemodialysis.

### Limitations

In this study, the only ELISA assay was performed on collected sera with no supporting data by molecular confirmation. As evident, it would have been ideal to perform PCR technique on IgM positive samples by ELISA, but this point was not possible due to financial constraints. On the other hands, the PCR technique is used when the patient is in the acute stage and the tachyzoites are in the bloodstream. Since this step is very short, therefore PCR only responds to patients who are positive for IgM and is mostly used for immunocompromised subjects.

## Data Availability

The datasets used and/or analysed during the current study available from the corresponding author on reasonable request.

## Abbreviations

ELISA: Enzyme-linked immunosorbent assay
HDP: Hemodialysis patients
IgG: Immunoglobulin G
IgM: Immunoglobulin M
*T. gondii*: *Toxoplasma gondii*
